# Supplementary material for: MetaRibo-Seq measures translation in microbiomes
Source: Nat Commun. 2020 Jun 29;11:3268. doi: 10.1038/s41467-020-17081-z (PMC7324362; doi:10.1038/s41467-020-17081-z)
Supplement: Supplementary file 10 — Supplementary Data 7 [file 41467_2020_17081_MOESM10_ESM.zip › File2/Confidence_High_Taxonomy/133451_out.krona.html]

Javascript must be enabled to view this page.

members
magnitude
magnitudeUnassigned
count
unassigned
taxon
rank

133451\_out

68

superkingdom
68
2

68
1224
phylum

1236
68
class

135615
68
order

family
68
868

68
genus
2717

68
species

SRS013170\_contig\_number\_contig-100\_13634.161979SRS014894\_contig\_number\_contig-100\_12002.25366SRS015064\_contig\_number\_52SRS016474\_contig\_number\_contig-100\_2724.179586SRS016746\_contig\_number\_contig-100\_16285.94188SRS016876\_contig\_number\_28280SRS017227\_contig\_number\_66885SRS017511\_contig\_number\_contig-100\_10066.156331SRS017757\_contig\_number\_35343SRS018061\_contig\_number\_contig-100\_1130.45878SRS018826\_contig\_number\_contig-100\_17722.150154SRS018975\_contig\_number\_19030SRS019077\_contig\_number\_contig-100\_9100.46679SRS019128\_contig\_number\_24317SRS019129\_contig\_number\_contig-100\_7685.87902SRS019494\_contig\_number\_contig-100\_2038.65765SRS021477\_contig\_number\_24968SRS022149\_contig\_number\_22103SRS022536\_contig\_number\_42141SRS023595\_contig\_number\_68917SRS023841\_contig\_number\_contig-100\_5737.63545SRS024087\_contig\_number\_37461SRS024355\_contig\_number\_contig-100\_14335.93842SRS042984\_contig\_number\_41977SRS043018\_contig\_number\_22487SRS043729\_contig\_number\_contig-100\_6252.68293SRS045588\_contig\_number\_contig-100\_11241.46529SRS045763\_contig\_number\_contig-100\_13137.60714SRS047113\_contig\_number\_contig-100\_4831.228544SRS047634\_contig\_number\_36466SRS058808\_contig\_number\_15514SRS065099\_contig\_number\_19879SRS074598\_contig\_number\_8685SRS075410\_contig\_number\_20245SRS075993\_contig\_number\_contig-100\_398.57891SRS076821\_contig\_number\_19671SRS077245\_contig\_number\_25850SRS077447\_contig\_number\_contig-100\_2327.103424SRS078223\_contig\_number\_25621SRS078431\_contig\_number\_contig-100\_867.173430SRS078671\_contig\_number\_contig-100\_14043.151587SRS078738\_contig\_number\_24929SRS097871\_contig\_number\_14884SRS1041121\_contig\_number\_contig-100\_710.94783SRS104177\_contig\_number\_14191SRS104320\_contig\_number\_30877SRS104921\_contig\_number\_contig-100\_2264.115001SRS105074\_contig\_number\_23101SRS1055033\_contig\_number\_contig-100\_11409.76274SRS142551\_contig\_number\_16805SRS143094\_contig\_number\_contig-100\_2851.65873SRS143885\_contig\_number\_contig-100\_5604.171188SRS144096\_contig\_number\_26549SRS144402\_contig\_number\_contig-100\_11768.46370SRS146773\_contig\_number\_25374SRS147057\_contig\_number\_11158SRS147106\_contig\_number\_contig-100\_8339.238621SRS147128\_contig\_number\_33544SRS147386\_contig\_number\_47658SRS147547\_contig\_number\_21661SRS148193\_contig\_number\_contig-100\_6782.312941SRS148205\_contig\_number\_4271SRS148826\_contig\_number\_5393SRS148979\_contig\_number\_11921SRS149286\_contig\_number\_22533SRS893281\_contig\_number\_contig-100\_14171.87729SRS893283\_contig\_number\_contig-100\_9939.42043SRS893347\_contig\_number\_5554
2718
